# Comparative Transcriptome Sequencing and Endogenous Phytohormone Content of Annual Grafted Branches of *Zelkova schneideriana* and Its Dwarf Variety HenTianGao

**DOI:** 10.3390/ijms242316902

**Published:** 2023-11-29

**Authors:** Chenfei Huang, Xiaoling Jin, Haiyan Lin, Jinsong He, Yan Chen

**Affiliations:** 1 College of Landscape Architecture, Central South University of Forestry and Technology, Changsha 410004, China; t20100192@csuft.edu.cn (C.H.); hjs1802893476@126.com (J.H.);; 2 Hunan Big Data Engineering Technology Research Center of Natural Protected Areas Landscape Resources, Changsha 410004, China; 3 College of Horticulture, Hunan Agricultural University, Changsha 410128, China

**Keywords:** auxin, cytokinin, jasmonic acid, plant height, stem elongation, cell size, cell wall

## Abstract

*Zelkova schneideriana* is a fast-growing tree species endemic to China. Recent surveys and reports have highlighted a continued decline in its natural populations; therefore, it is included in the Red List of Threatened Species by The International Union for Conservation of Nature. A new variety “HenTianGao” (H) has been developed with smaller plant height, slow growth, and lower branching points. In this study, we attempted to understand the differences in plant height of *Z. schneideriana* (J) and its dwarf variety H. We determined the endogenous hormone content in the annual grafted branches of both J and H. J exhibited higher gibberellic acid (GA)-19 and trans-Zeatin (tZ) levels, whereas H had higher levels of indole-3-acetic acid (IAA) catabolite 2-oxindole-3-acetic acid (OxIAA), IAA-Glu conjugate, and jasmonic acid (JA) (and its conjugate JA-Ile). The transcriptome comparison showed differential regulation of 20,944 genes enriched in growth and development, signaling, and metabolism-related pathways. The results show that the differential phytohormone level (IAA, JA, tZ, and GA) was consistent with the expression of the genes associated with their biosynthesis. The differences in relative OxIAA, IAA-Glu, GA19, trans-Zeatin, JA, and JA-Ile levels were linked to changes in respective signaling-related genes. We also observed significant differences in the expression of cell size, number, proliferation, cell wall biosynthesis, and remodeling-related genes in J and H. The differences in relative endogenous hormone levels, expression of biosynthesis, and signaling genes provide a theoretical basis for understanding the plant height differences in *Z. schneideriana*.

## 1. Introduction

*Zelkova schneideriana* is a member of the *Ulmaeae* family. It is a large and fast-growing tree native to Asia. It is an endemic species in China, where it is mainly distributed in southern China. It is used as a commercial wood for furniture, boats, and wooden bridges. Due to the large-scale cut-down, it has been categorized as a second-class national key protected plant in China. The International Union for Conservation of Nature (IUCN) has included this species in the Red List of Threatened Species [1]. Therefore, continued efforts are needed to develop new germplasm, improve the existing varieties through modern plant breeding and genomics techniques, and to conserve the species, as it faces the possibility of population loss due to limited expansion into suitable areas. The simulations-based study showed that even under human management, significant efforts will be needed to protect and expand its distribution in China [2].

In 2016, a new variety of *Z. schneideriana*, HenTianGao (H), was bred through mutation breeding. This variety has shown relatively small plant height (1.5 m height), slow growth, low branching points, late autumn leaf color change, and late leaf fall. All these characteristics are heritable and the survival rate of its grafted populations is high, thus making it a suitable tree for landscaping [3]. These characteristics differ from those of naturally distributed *Z. schneideriana* trees (up to 35 m height, J). The observable differences in growth, plant height, and branching points in J and H indicate the possibility of different growth regulation mechanisms. To date, only limited data are available, as the major focus of the research has been on in-vitro regeneration [4], evolution [5], and the development of molecular markers for genetic diversity studies [6,7]. However, some earlier investigations on other seedlings and saplings of *Z. schneideriana* have shown that the trees can differ in growth traits and such traits are moderately to highly heritable [8]. Understanding the mechanism of differential growth can help conservationists and breeders develop varieties with fast growth and better survival. Therefore, using H as an experimental plant, we aimed to compare its annual grafted branches with those of normal *Z. schneideriana* trees (J) to explore the candidate pathways involved in differential growth.

Plant height is an important trait that contributes to biomass and yield. Multiple factors have been associated with the differential stem height in different plant species. Notable factors include the biosynthesis and signaling of phytohormones, e.g., gibberellic acid (GA) [9], auxin [10,11], jasmonic acid (JA) [12], cytokinin, and ethylene [13], cell size, number of cells [14,15,16,17,18], and the ability of the cell wall to expand and remodel [19]. Among the hormones, GAs have been implicated in stem elongation by affecting cell elongation and division [20] through the induction of genes involved in GA signaling and cell wall biosynthesis/remodeling. For example, xyloglucan endotransglycosylases as well as expansins are GA-responsive cell-wall-related genes in rice, wheat, cabbage, and Arabidopsis [21,22,23,24]. Plant cell growth is achieved by a regulated balance between the ability of the cell wall to expand (extensibility) and forces exerted on the cell wall such as turgor pressure [25]. Auxins also elongate stems by promoting cell growth. This cell growth is achieved by increasing cell wall extensibility and loosening [26]. A strict balance between cell wall rigidity and flexibility is imperative for plant growth with differential stem length or plant height [27]. Such changes in plant cell walls are due to a diversity of cell wall biosynthesis, rearrangement, and loosening-related genes [28,29]. The cell-wall modifying/biosynthesis genes are regulated by auxin signaling, e.g., different AUX/IAA Arabidopsis mutants exhibit defects in cell expansion, indicating an important role of auxin in cell expansion [30]. Similarly, the auxin response factor (ARF) can positively regulate the expressions of expansins [31]. Other than GA and auxin, cytokinins also play a key role in stem elongation [32]. Cytokinin-responsive growth regulators control cell expansion and cell-cycle progression, contributing to better growth [33]. Contrary to GA, auxin, and cytokinin, JA has been shown to repress several growth aspects, e.g., hypocotyl elongation [34]. The JA-GA, JA-auxin, and JA-cytokinin can have antagonistic effects on many aspects of plant growth, e.g., root growth, leaf senescence, xylem development, chlorophyll development, etc. [12]. Thus, for plants differing in height, internode length, or stem elongation, one can expect the differential accumulation of these hormones and related signaling genes’ expression.

To gain a preliminary understanding of the potential causes of the differential heights of the annual grafted branches of J and H, we used transcriptome sequencing and endogenous phytohormone-level determination by the UPLC-ESI-MS/MS system. Phytohormones are essential for controlling plant growth and development. Metabolism provides both the building blocks and the energy for survival, whilst hormones regulate plant development. Therefore, we report the differential phytohormone profiles (auxins, cytokinins, JA, and GA) and expression changes in their biosynthesis and signaling in J and H. Moreover, we also examined variations in the expression of genes/transcription factors involved in phytohormone biosynthesis and signaling, as well as those involved in stem elongation, cell size, cell number, cell proliferation, and cell wall biosynthesis. This report will lay the foundation for the next steps of functional characterization of genes related to differential growth.

## 2. Results

### 2.1. Growth Form and Histological Observations

**Figure 1:**
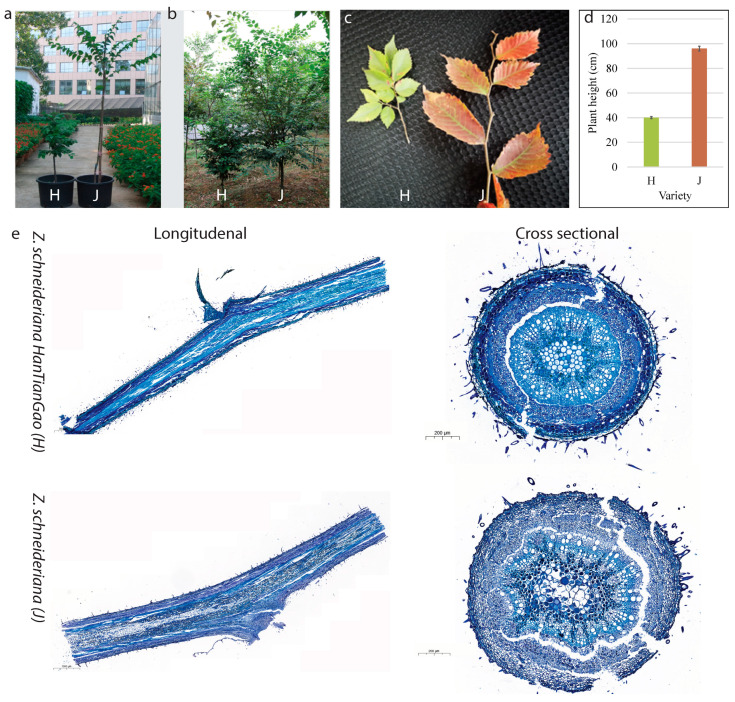
Variations in growth habits of *Z. schneideriana* ‘HenTianGao’ (H) and *Z. schneideriana* (J). (**a**) Overview of the differential growth habits of (**a**) one-year-old and (**b**) three-year-old plants, and (**c**) branches of mature trees. (**d**) Average plant height (*n* = 30) of H and J. The error bars show the standard deviation. (**e**) Light microscope sections (cross-section and longitudinal) of *Z. schneideriana* showing internal organ cell size variations. Larger cell sizes are visible in J compared to H.

The *Z. schneideriana* (J) and *Z. schneideriana* HenTianGao (H) show observable variations in growth forms such that J has longer stems with larger internodes compared to H (Figure 1a–c). It can be seen from Figure 1d that the average plant height of J is 96.05 cm compared to that of H (40.06). The histological comparison of J and H stem sections shows that the cells in J tend to be larger than those in H (Figure 1e).

### 2.2. Phytohormone Profiles of Z. schneideriana and HenTianGao

**Figure 2:**
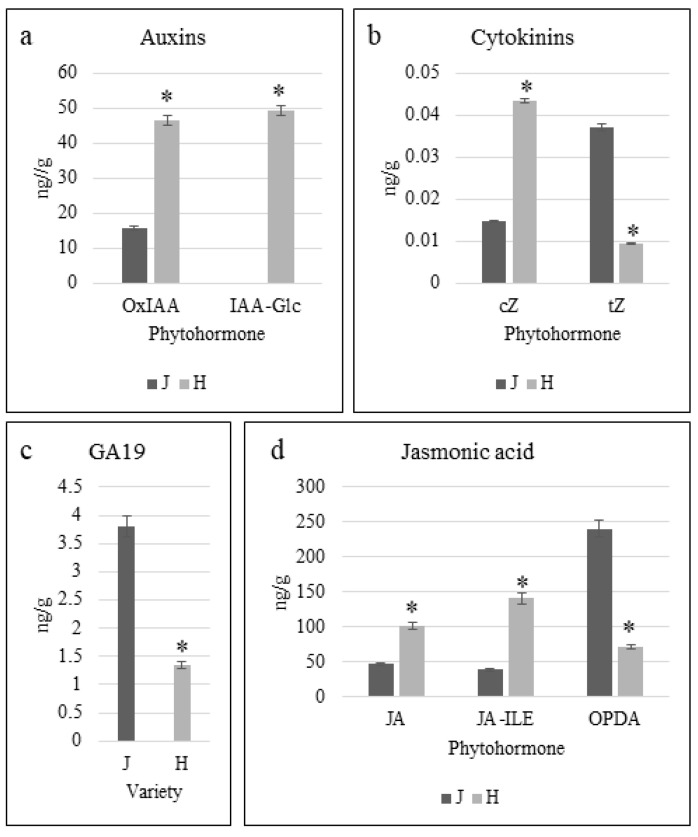
Endogenous phytohormone levels (**a**) indole-3-acetic acid conjugates, (**b**) cytokinins, (**c**) gibberellic acid 19, and (**d**) jasmonic acid and its conjugates in *Z. schneideriana* ‘HenTianGao’ (H) and *Z. schneideriana* (J) one-year-old stems. Error bars show standard deviation. * shows that the differences are significant at *p* (0.05). The axis shows the phytohormone compound and the *y*-axis shows the relative intensity (average of three replicates) of each phytohormone.

The endogenous levels of auxins (2-oxindole-3-acetic acid, Ox-IAA and IAA-glucoside, IAA-Glc), cytokinins (BAP7G, cZ, tZ, and BAP), gibberellic acid (GA19), and jasmonic acid (JA, JA-ILE, and OPDA) showed observable variations between the two varieties (Table S1). The levels of IAA-amino acid conjugates, Ox-IAA and IAA-Glc, were significantly higher in H as compared to J. Notably, IAA-Glc was only detected in H (Figure 2a). The higher levels of Ox-IAA and IAA-Glc suggest the possibility that the IAA in H is increasingly converted to these conjugates compared to J. In the case of cytokinins, J had significantly higher tZ levels compared to H, whereas the cis-zeatin (cZ) levels showed an opposite accumulation trend (Figure 2b). The GA19 levels were significantly lower in H as compared to J (Figure 2c). The JA and JA-Ile levels were significantly higher in H as compared to J. Whereas, the biosynthetic precursor of JA, oxylipin 12-oxo-phytodienoic acid (OPDA) levels, corresponded well with JA and JA-Ile levels, OPDA levels were significantly lower in H as compared to J (Figure 2d). Taken together, it was observed that the auxin, cytokinin (BAP7G and cZ), and JA levels were higher in H as compared to J.

### 2.3. Transcriptome Sequencing of Z. schneideriana and HenTianGao

**Figure 3:**
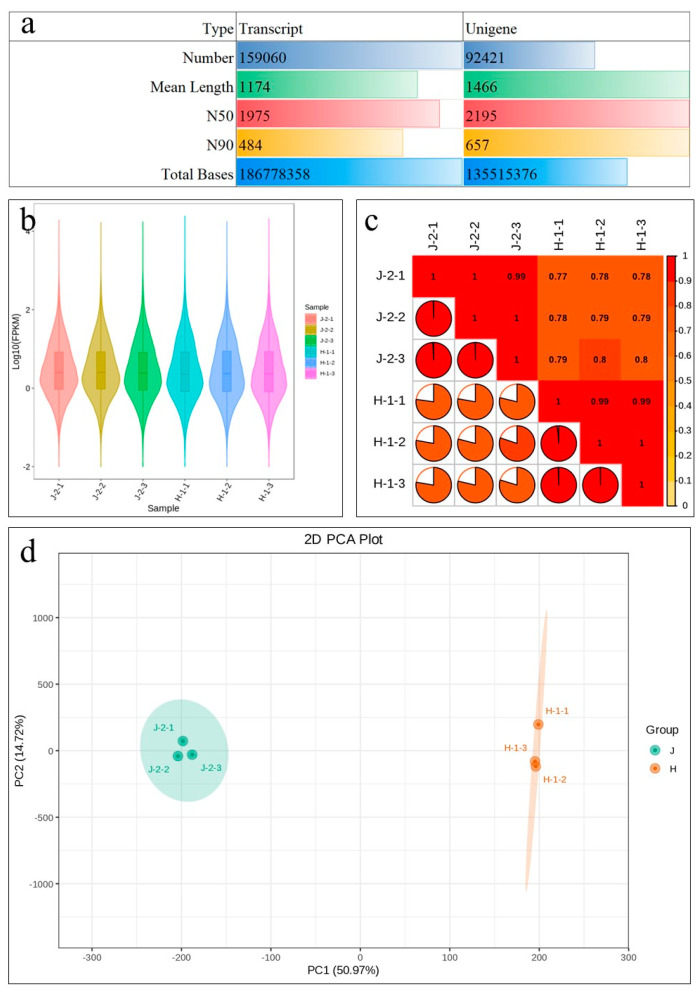
Summary of transcriptome sequencing of *Z. schneideriana* stem cutting. (**a**) Total transcript splicing results statistics, (**b**) overall distribution of gene expression (FPKM), (**c**) Pearson’s Correlation Coefficient, and (**d**) Principal Component Analysis based on FPKM values of J and H. The numbers with the sample type indicate the replicates.

The sequencing of six libraries produced, on average, 52,112,940 raw reads per library. After filtering out low-quality reads, 46.94 Gb of clean data was obtained, for which the Q20%, Q30%, error rate (%), and GC content (%) were 98%, 94%, 0.03%, and 44% (Table S2). The transcript assembly by Trinity was used for subsequent analyses. After hierarchical clustering by Corset, the longest cluster sequence was obtained as unigene for further analysis. In total, the sequencing produced 159,060 transcripts and 92,421 unigenes (Figure 3a). At least 71,682 (77.56%) genes could be annotated (Figure S1). Comparison of the clean reads with the assembled transcript sequences, resulted in an average of 85% of the reads that could be mapped (Table S2). Overall gene expression (FPKM) of the H was lower than J (Figure 3b). The Pearson’s Correlation Coefficient between the varieties (replicates) was ~0.79 (Figure 3c). The Principal Component Analysis (PCA) showed the groping of replicates of each variety (Figure 3d). These statistics indicate that the sequencing results are reliable.

### 2.4. Differential Gene Expression between Z. schneideriana and HenTianGao

**Figure 4:**
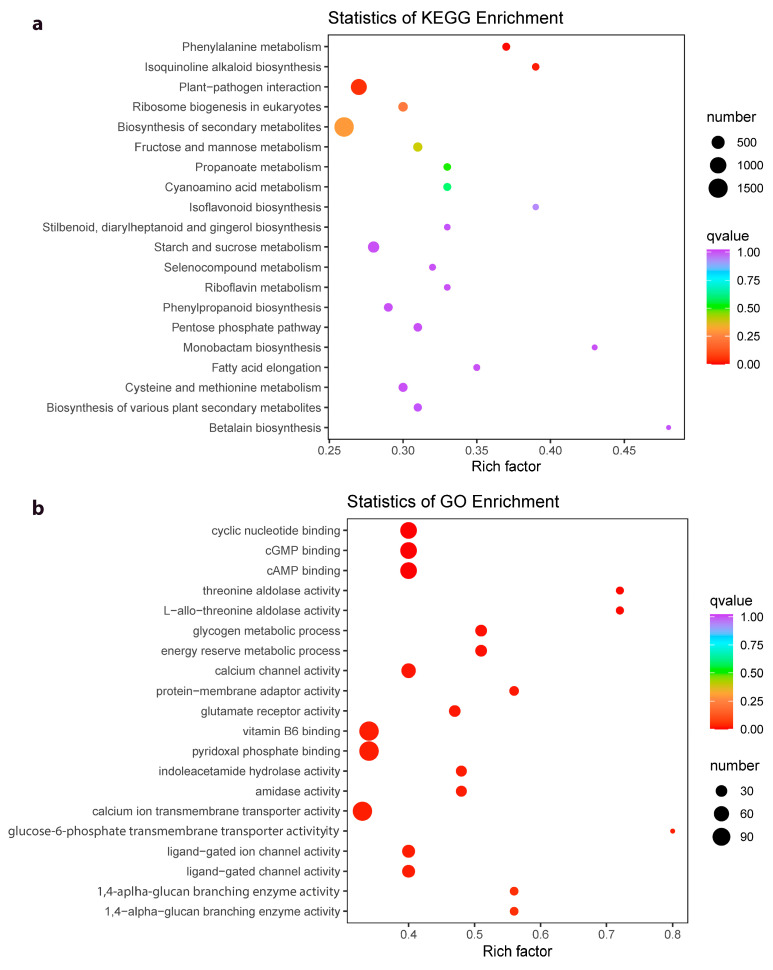
Scatter plots of the (**a**) KEGG and (**b**) GO pathways to which the differentially expressed genes in J and H were significantly enriched.

The screening conditions, |log2 fold change| ≥ 1 and False Discovery Rate (FDR) < 0.05, resulted in the identification of 20944 differentially expressed genes (DEGs). The DEGs were enriched in 143 KEGG pathways (Figure S2a) and 5538 GO terms (cellular component, biological process, and molecular function) (Figure S2b). The KEGG pathways to which the DEGs were significantly enriched included phenylalanine metabolism, isoquinoline alkaloid biosynthesis, plant-pathogen interaction, ribosome biogenesis in eukaryotes, biosynthesis of secondary metabolites, and fructose and mannose metabolism (Figure 4a). On the other hand, the GO pathways in which the DEGs were significantly enriched include several binding terms (e.g., cyclic nucleotide, cGMP, and cAMP binding), enzyme activities (calcium channel, protein-membrane adaptor, glutamate receptor, threonine aldolase, and protein-membrane adaptor activities), and processes (glycogen metabolic process, energy reserve metabolic process, etc.) (Figure 4b).

There were 4565 and 2216 genes exclusively expressed in J and H, respectively. Those expressed in J and H were enriched in 204 and 180 KEGG pathways, respectively; 175 of these KEGG pathways were common (Table S3). The DEGs with the highest log2 FC (expressed exclusively in J) include cellulose synthase A (CESA, *Cluster-36852.4*), phytepsin (*Cluster-35274.7*), tRNA guanosine-2′-O-methyltransferase (*Cluster-32814.0*), catalase (*Cluster-34143.5*), and fumarylacetoacetase (*Cluster-28498.2*). The DEGs with the lowest log2 FC (expressed exclusively in H) include 1,4-alpha-glucan branching enzyme (Cluster-33083.3), tRNA guanosine 2′-O-methyltransferase (Cluster-32814.2), phospholipase D (Cluster-28941.2), phytepsin (Cluster-35274.2), and CESA (Cluster-36852.0). The exclusive expression of genes with the same annotation and their enrichment in 175 common KEGG pathways indicate the possibility that the two varieties may express different transcripts of the same genes enriched in the same pathways.

### 2.5. Transcriptome Sequencing Confirm the Phytohormone Accumulation Trends

**Figure 5:**
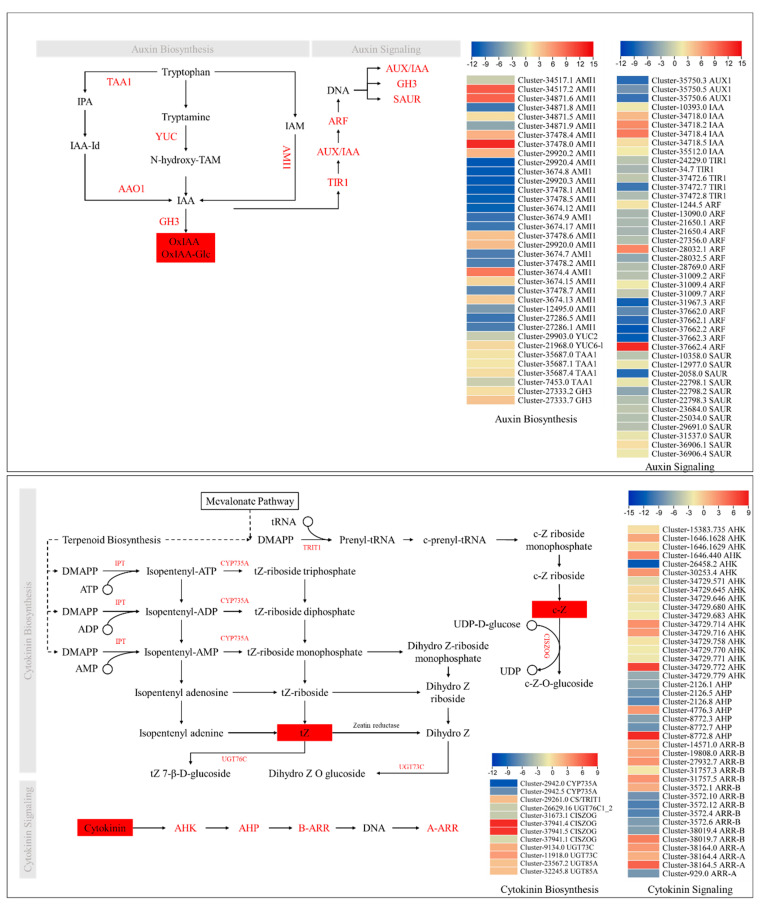
Differential regulation of the auxin and cytokinin biosynthesis and signaling pathways in J vs. H. The genes in red are the DEGs. The compounds (hormones) in red boxes were differently accumulated in J vs. H. The heatmaps show log2 fold change values of the DEGs in J vs. H.

As we noticed significant changes in the accumulation of IAA conjugates, cytokinins, GA19, and JA, we searched for the expression changes in the related genes. Seventeen of the 28 amidase (AMI1) transcripts had higher expressions in J as compared to H. AMI1 converts indole-3-acetamide to IAA. Interestingly, 12 AMI1s were exclusively expressed in J, whereas only four AMI1s were exclusively expressed in H. This expression suggests that IAA biosynthesis maybe higher in J than in H. On the other hand, three L-tryptophan—pyruvate aminotransferase 1 (TAA1) transcripts, which convert tryptophan (Trp) to tryptamine (TAM), had higher expressions in H compared to J. One TAA1 (*Cluster-7453.0*) had higher expression in J compared to H. Next, TAM is converted into N-hydroxy-TAM (HTAM) by the action of indole-3-pyruvate monooxygenase YUCCA (YUC). A YUC6-like gene had higher expression in H as compared to Y, whereas a YUC2 had higher expression in J compared to H. The expression of YUC6-like and YUC2 indicates that both J and H may use different sets of YUCs for auxin biosynthesis via the indole-3-pyruvic acid (IPA) pathway. Finally, the higher expression of the two GH3s (*Cluster-27333.2* and *Cluster-27333.7*) (Figure 5) further strengthens our proposition that in H, the IAA is increasingly converted to IAA conjugates because GH3s are IAA-amino acid conjugating enzymes, convert IAA to its catabolite 2-oxindole-3-acetic acid (OxIAA) and amino acid conjugate OxIAA-Gluc [35]. These expressions are consistent with the higher auxin conjugates’ levels in H as compared to J (Figure 2). Taken together, the expressions and endogenous OxIAA and IAA-Glc levels indicate higher conversion of IAA to its amino acid conjugates, hence IAA overaccumulation is possibly restricted in H.

Regarding auxin signaling, the auxin influx carriers (AUX1s), transport inhibitor response 1s (TIR1s), and most of the auxin response factors (ARFs) had higher expressions in J compared to H. The auxin-responsive protein IAA (AUX/IAA) transcripts had higher expressions in H than in J, whereas, the SAUR family proteins (SAURs) exhibited variable expression patterns (Figure 5). Therefore, the increased conversion of IAA to its amino acid conjugates in H could be a reason for AUX1 and ARF expressions.

For cytokinin biosynthesis, we observed the expression patterns of the genes enriched in zeatin biosynthesis. The cytokinin trans-hydroxylase (CYP753A1/A2), cytokinin-N-glucosyltransferase (CYTGtf), and cis-zeatin O-glucosyltransferase (cisZOG) had higher expressions in J compared to H. Whereas, UDP-glucosyltransferase 73C (UGT73C), UCP-glucosyltransferase 85A2 (UGT85A2), and cytokinin synthase (CS, *Cluster-29261.0*) had lower expressions in J compared to H. The GO annotation of CS also showed the presence of the term “tRNA dimethylallyltransferase, TRIT1”. TRIT1 converts DMAPP to prenyl-tRNA, which is then converted to cZ through three intermediates [36]. The expressions of CS (or TRIT1 and cisZOG (*Cluster-37941.4* and *Cluster-37941.5*) are consistent with the higher cZ level in H. Whereas, the higher expression of CYP735A (*Cluster-2942.0* and *Cluster-2942.2*) is consistent with higher tZ level in J than in H. Another reason for the lower tZ level in H could be the higher expression of UGT73C/UGT85A1, which convert dihydro zeatin to dihydrozeatin-O-glucoside [37] (Figure 5).

Furthermore, we checked the differential expression of cytokinin-signaling-related genes between J and H. The Arabidopsis histidine kinase (AHK) and the two-component response regulator ARR-B transcripts showed variable expression patterns. Most of the histidine-containing phosphotransfer proteins (AHPs) were exclusively expressed in J except for two transcripts (*Cluster-4776.3* and *Cluster-8772.8*), which showed exclusive expressions in H. The two-component response regulator ARR-As were mostly highly expressed in H, except for one that was exclusively expressed in J (Figure 5).

**Figure 6:**
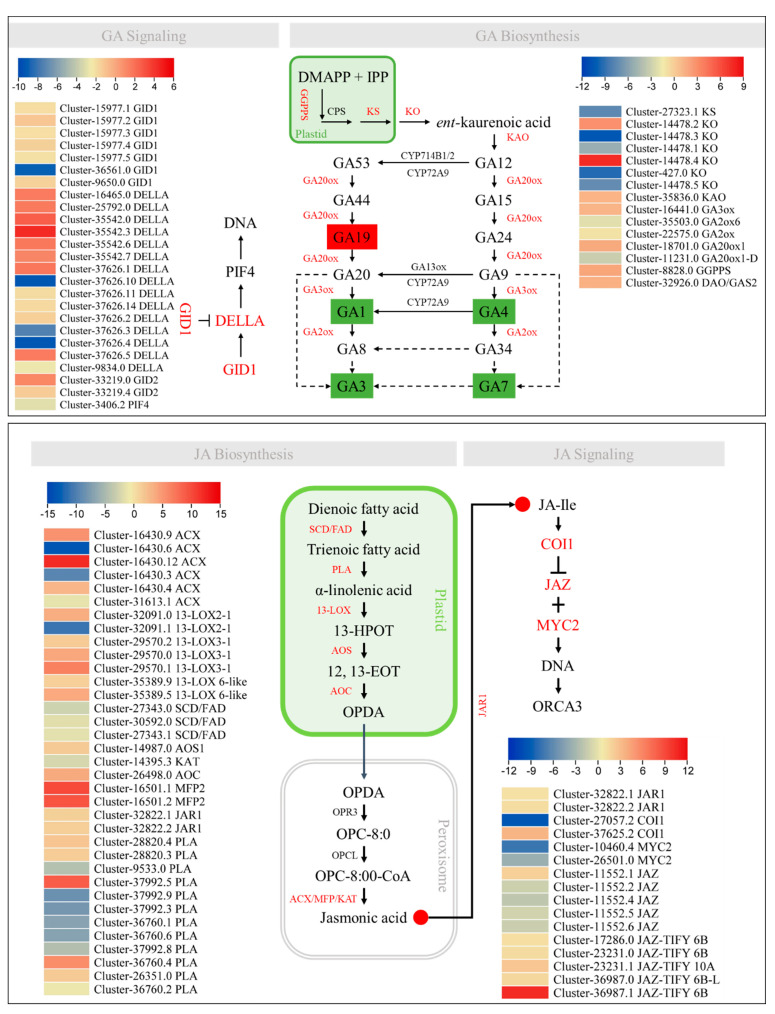
Differential regulation of GA and JA biosynthesis and signaling pathways in J vs. H. The genes in red are the DEGs. The compounds (hormones) in red boxes/circles were differently accumulated in J vs. H. The GAs shown in green are the active GAs. The heatmaps show log2 fold change values of the DEGs in J vs. H.

The GA19 level was higher in J than H. GA19 is converted to GA20 by the action of GA20ox, which is further converted to either GA1 or GA3 by the action of GA3ox. The GA3ox and GA20ox1 had higher expressions in H than J, whereas, two GA2ox and one GA20ox1-D had higher expressions in J than H. In addition, several upstream genes such as geranylgeranyl diphosphate synthase (GGPPS), ent-kaurene synthase (KS), ent-kaurene oxidase (KO), ent-kaurenoic acid oxidase 2-like (KAO), and 2-oxoglutarate-dependent dioxygenase (DAO-like or GAS2) were also differentially expressed. The GGPPS, KAO, and DAO-like had higher expressions in H than in J, whereas the others showed the opposite expression trends. The increased accumulation of G19 and the higher expressions of GA2ox and GA20ox1-D suggest the possibility of a more active GA biosynthesis in J than in H. This statement is further supported by the expression of genes related to GA signaling. The gibberellin receptor GID1 and phytochrome-interacting factor 4 (PIF4) had higher expressions in J than H. The F-box GID2 and DELLA proteins showed variable expression trends, i.e., some had higher expressions in H and the others had higher expressions in J (Figure 6).

Finally, we checked the expression differences in the two varieties for JA biosynthesis and signaling. Three stearoyl-CoA desaturase (SCD/FAD) and acetyl-CoA acyltransferase (KAT) had lower expressions in H than in J. The phospholipase A1s (PLAs), 13-lipoxygenase 2-1s (13-LOX 2-1), and acyl-CoA oxidases (ACXs) had variable expressions. Whereas, the 13-LOX 3-1, 13-LOX 6-like, allene oxide synthase 1 (AOX1), allene oxide cyclase (AOC), multifunctional proteins (or anoyl-CoA hydratase/3-hydroxyacyl-CoA dehydrogenases (MFP2s), and jasmonic acid-amino synthetase (JAR1) had higher expressions in H than in J. The JA-signaling-related genes MYC2 were exclusively expressed in J, while the jasmonate ZIM domain-containing proteins (JAZ, protein TIFY 6B, 6B-like, and 10A) and JAR1s had higher expressions in H than in J. Contrastingly, some JAZs showed contrasting expressions too. Similarly, the coronatine-insensitive protein 1 (CO1) and showed variable expression patterns (Figure 6).

### 2.6. Expression Changes in Genes Related to Stem Elongation, Cell Size, Number, and Proliferation

**Figure 7:**
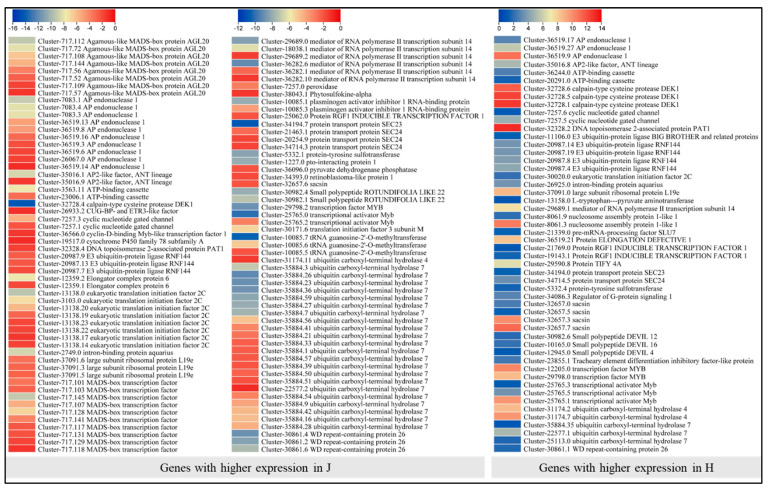
Heatmaps of log2FC value of cell-size-related genes in *Z. schneideriana* ‘HenTianGao’ (H) and *Z. schneideriana* (J) one-year-old stems.

A search using the GO terms “cell number”, “cell size”, and “cell proliferation” resulted in screening 163 transcripts; 110 were highly expressed in J compared to H, while the remaining 53 were highly expressed in H. The transcripts that were highly expressed in J were annotated as MADS-box transcription factors (MADS-TF), elongation complex proteins, AGAMOUS-like 42, wall-associated receptor kinases (WAK), ribosomal proteins (L19e), cyclic nucleotide gated channel (CNGC), ATP-binding cassette, AP2-like ethylene responsive TF, transcriptional activator Myb, AP endonuclease 1, eukaryotic translation initiation factor 2C, ubiquitin carboxyl-terminal hydrolase 7, E3 ubiquitin-protein ligase RNF144, DNA topoisomerase 2-associated protein PAT1, protein transport protein SEC23 and SEC24, mediator of RNA polymerase II transcription subunit 14, tRNA guanosine-2′-O-methyltransferase, WD repeat-containing protein 26, and some other genes (Figure 7). These observations indicate that a relatively diverse set of genes belonging to different gene families and functional annotations are highly expressed in J. The Agamous-like MADS-box protein AGL20 is associated with cell differentiation, whereas, CNGCs have been implicated in programmed cell death [38]. The ATP-binding cassettes have multiple functions including continuous growth of tip-growing cells by modulating reactive oxygen species (ROS) generation and shootward movement of metabolites, etc., [39]. Similarly, WD repeat-containing proteins take part in organ-size variations [40]. Thus, the higher expressions of the above-mentioned genes could be linked with the morphology of J compared to H. Contrarily, several transcripts associated with AP endonuclease 1, calpain-type cysteine protease DEK1, CNGCs, RNF144, Protein ELONGATION DEFECTIVE 1 (ELD1), small polypeptide DEVIL 12/16/4, and others had higher expressions in H (Figure 7). Overall, we observed large-scale expression differences for genes related to cell size in J and H. These are the ideal candidates for future functional characterization and gene manipulation studies related to cell size in this or related species.

Additionally, we searched for genes that have been reported for their role in cell size and stem elongation [14,15,16,17,18]. There were 72 DEGs associated with these functions, either negative regulators of cell proliferation or positive regulators; 48 had higher expression in J compared to H, while the remaining genes had higher expression in H. Interestingly, 25 of the 72 DEGs were exclusively expressed in J. These included B3-domain-containing TF VRN1 (VRN1), cell number regulator 6 (CNR6), PEAPOD2, erine/threonine-protein kinase mTOR, AP2-like factor, cinnamoyl-CoA reductase (CCOAR), erbb2-interacting protein, and BIG BROTHER (BB). The genes that showed higher expression in J included CCOAR, erbb2-interacting protein, target of rapamycin complex subunit LST8, VRN1, retinoblastoma-like protein 1 (RBL1), mTOR, E3 ubiquitin-protein ligase RNF38/44, and PEAPOD2. Notably, there was a relatively lower number of upregulated transcripts of VRN1, mTOR, CCOAR, erbb2, RNF38/44, BB, and PEAPOD1/2. The larger number of transcripts with higher expressions in J is consistent with its longer stems. Among these, PEAPOD and BB are negative regulators of cell proliferation. Their differential expression indicates that both varieties have genes that control cell proliferation (Table S4).

### 2.7. Differential Expression of Genes Related to Cell Wall

There were 696 transcripts annotated with the GO term “cell wall”, of which 364 were highly expressed in J compared to H. The 696 transcripts were annotated as 190 different genes (families). The genes that were highly expressed in J than in H were 1,4-beta-D-xylan synthase, basic endochitinase B, brassinosteroid insensitive 1-associated receptor kinase 1, COBRA-like proteins, elongation factor Tu, galacturonosyltransferase 12/13/14/15, and small subunit ribosomal protein S10e/S18e/S3Ae/S9e. Overall, we observed that the number of highly expressed transcripts was higher in J compared to H for receptor-like protein kinase FERONIA, Protein TRACHEARY ELEMENT DIFFERENTIATION-RELATED 7A, phosphatidylinositol 3,5-bisphosphate 5-phosphatase, pectinesterase, microtubule-associated protein 70-5, mannan endo-1,4-beta-mannosidase, kinesin family members 7/4C, heparinase, and endoglucanases. Whereas the other cell-wall-related genes such as cellulose synthase A, beta-glucosidase, auxin efflux carrier family protein, AP endonuclease 1, alpha-mannosidase, and expansins showed mixed regulation. Interestingly, 152 cell-wall-related genes were exclusively expressed in J compared to 85 in H. These differences in expression indicate that both varieties have different cell-wall-related transcripts that could be regulating differential cell-wall biosynthesis and modification (Table S5).

### 2.8. Differential Expression of Transcription Factors

Transcription factors play an important role in plant growth and development. There were 734 differentially expressed TFs in J vs. H. The highest number of TFs were classified as NAC (38), followed by AP2/ERF-ERF (37), MYB-related (34), MYB (28), and C3H (26). Overall, NAC, AP2/ERF, MYB-related, MYB, C2H2, C3H, WRKY, bZIP, GARP-G2-like, bHLH, SNF2, GRAS, and Tify had a greater number of transcripts with higher expression in H compared to J. Whereas, B3, SET, MADS-G2-like, PHD, SBP, GNAT, Jumonji, SW1/SNF-BAF60b, NF-YA, TRAF, GRF, C2C2-GATA, and others had a greater number of transcripts that showed higher expression in J compared to H (Table S6). These TFs were enriched in several pathways related to development, signal transduction, and metabolism such as circadian rhythm, peroxisomes, endocytosis, protein processing in endoplasmic reticulum, plant hormone signal transduction, MAPK signaling, homologous recombination, base excision repair, spliceosome, amino sugar, nucleotide sugar metabolism, and many amino acid biosynthesis/metabolism-related pathways (Table S6). These expressions indicate that the two varieties differ in terms of the number and type (up- and downregulated) transcription factors enriched in primary and secondary metabolism, plant growth and development, and signal transduction.

### 2.9. Validation of Gene Expression by qRT-PCR Analysis

**Figure 8:**
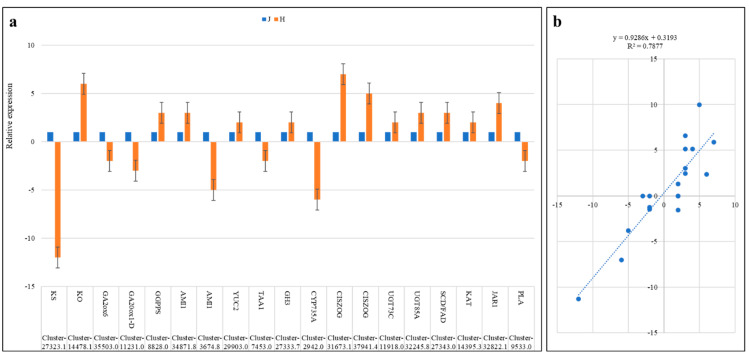
(**a**) QRT-PCR validation of *Z. schneideriana* genes in one-year-old J and H stems. The values are means (*n* = 3) ± SD. (**b**) Correlation between relative expression and FPKM of the selected genes.

We validated the expression profiles of 19 *Z. schneideriana* genes in J and H. The qRT-PCR analysis of the selected genes showed a similar expression trend as of RNA-sequencing-based FPKM values (Figure 8a). This was further confirmed by a positive correlation (R^2^ = ~0.79) between the two expression datasets (Figure 8b). These expressions confirm the reliability of the RNA sequencing and highlight the putative roles of these genes in the differential regulation of related pathways.

## 3. Discussion

*Zelkova schneideriana* grows in small populations in the mountainous areas of 16 Chines provinces, occupying ~560 km^2^. Its population in China has declined by 30 to 35% over the last 180 years in China due to overexploitation [1]. Such a decline is the result of extensive deforestation and demand for its wood. Its wood is very expansive due to its characteristics such as a dark reddish color, high quality, and decay-resistant wood [41]. The decline of subpopulations still continues; therefore, it is considered a Vulnerable A2c species. This problem further intensifies as the generation rate of this species is very low in its natural habitat. With the development of a new variety, HenTianGao, we have the opportunity to understand the differential regulation of plant height in J and H [3]. Here, we discuss the key differences in auxin, GA, cytokinin, and JA levels in one-year-old annual grafted branches of J and H. We also discuss the expression changes in phytohormone biosynthesis and signaling-related genes together with the genes related to stem elongation, cell size, cell number, cell proliferation, cell wall biosynthesis, and transcription factors.

Phytohormones play a critical role in controlling plant growth and development. Metabolism provides both the building blocks as well as energy for survival, whereas the hormones regulate the growth aspects of a plant [42]. Particularly, the stem elongation in plants is highly dependent on GAs [43]. Our observation that the GA19 level was higher in J than in H (Figure 2b), which suggests that the former has a higher GA biosynthesis than the latter. GA19 is converted to bioactive GAs (GA1 and GA3) by the action of GA3ox and GA2ox [44]. Higher GA19 levels in J may indicate a relatively higher level of active GA biosynthesis than in H. These observations are also consistent with the increased expression of GID1 and PIF4. GID1 is a GA receptor and has been reported to positively affect stem elongation in *Jasminum sambac* [45]. Similarly, PIF4 interacts with blue light receptors and mediates hypocotyl elongation [46]. Thus, we can expect a similar role of GA and signaling in the differential plant height of J and H. In addition to GA, auxins also promote stem elongation by promoting cell growth. Furthermore, auxins also interact with GAs in such a way that the former promotes the biosynthesis of the latter, which then expands internodes [47]. The observations that OxIAA and IAA-Glu levels were significantly higher in H than in J indicate that IAA could be increasingly converted to their conjugates by the action of GH3s [35]. IAA is rapidly converted to its conjugates in response to increased IAA levels. Furthermore, OxIAA has little biological activity as observed in Arabidopsis [48]. Thus, the higher OxIAA and IAA-Glu in H could be an indication of lower IAA activity compared to J because IAA-amino acid conjugates are storage forms of IAA and can be converted back to IAA [49]. This proposition is consistent with the known role of auxin signaling genes AUX1, TIR1, and ARFs [50]. In addition to auxin signaling, the ARFs have also been implicated in hypocotyl elongation, cell division, and organ size [51,52]. Therefore, our results suggest that IAA is increasingly converted to OxIAA and IAA-Glu compared to J and IAA biosynthesis and signaling plays a role in the differential plant height and internode length in J and H. Other than IAA and GA, cytokinins control several growth and development-related aspects such as cell proliferation in plants. Zeatin is the most abundant form of cytokinins in higher plants; *trans*-Zeatin (tZ) is the active form and *cis*-Zeatin (cZ) is the less active form [53]. Here it is important to mention that the role of cZ in plant growth, abiotic and biotic stress tolerance, and herbivore resistance has been studied in multiple plant species [54]. Our observation that the active form (tZ) content was higher in J than in H indicates a possible correlation between plant height and tZ content. An earlier study has revealed that tZ controls the leaf size in maize [55], establishing a correlation between tZ and organ size. The increased accumulation of tZ in J is consistent with the observed differential expression (Figure 5) and the known role of zeatin biosynthesis genes [56]. Particularly, the increased expression of CYTGtf is indicative of a relatively active cytokinin homeostasis in J compared to H [57]. Contrarily, the higher content of cZ in H compared to J suggests that in the former variety, cZ biosynthesis is higher, which could be a possible cause of its differential height and internode length. These datasets are an important starting point for understanding the link between auxins, GA, and tZ in plant height and internode length in *Zelkova* species. Moreover, these findings are useful for the plant species in which the stem size is a limitation for agronomic practices.

Exogenous application of JA has been shown to negatively affect various growth aspects, including primary root growth, stem/hypocotyl elongation, and expansion of leaves [58]. High JA levels in *Nicotiana attenuate* caused a reduction in GA biosynthesis and inhibition of stem growth [59]. Our data that GA levels were higher in J, while the JA levels (JA-Ile and JA) were lower in J compared to H, indicating the presence of such an antagonistic effect of these hormones in *Z. schneideriana.* The higher JA content in H and the higher expression of 13-LOX, AOX1, AOC, MFP2, and JAR1 (Figure 6) are consistent with the known roles of these genes in JA biosynthesis [60]. JA and cytokinins also show contrasting effects on plant growth, such that JA suppresses the procambium-specific cytokinin response, whereas cytokinin can nullify the effect of JA on xylem [61]. Our observations that active cytokinin (tZ) level and JA level showed contrasting accumulation trends in J and H are consistent with these reports. Hence, our preliminary data put forward the proposition that plant height in *Z. schneideriana* is under the control of cross-talk between JA and growth-promoting hormones (IAA, GA, and tZ). This is important for understanding the interaction of these hormones for stem growth in *Zelkova* species. Future research can target either individual hormones or study the combined effect of the exogenous application of these hormones and then characterize the candidate genes.

The hormone signal transduction, e.g., auxin, GA, and cytokinin, regulates several growth and developmental processes, such as cell enlargement, cell division, and stem growth [62]. These cell enlargement and division processes are different in different zones of different organs [63]. Our observations that a large number of DEGs with GO terms related to “cell number”, “cell size”, and “cell proliferation” had higher expression in J compared to H are consistent with the differential accumulation of auxins, GA, and cytokinins. Particularly, the higher expressions of AGAMOUS-like MADS-box TF in J are consistent with their known role in cell proliferation in Arabidopsis [64]. Similarly, the higher expressions of WAK [65], AP2-like [66], ATP-binding cassette [67], and WD-repeat containing proteins [40] can be associated with the higher internode length and plant height in J compared to H, based on their known functions. Also, the higher expression of CNGCs in J suggests a potential role of calcium signaling in the differential plant height, as they are the main effectors in plants cells [68]. However, the relationship of calcium signaling with phytohormones discussed here would require further investigation. The exclusive expression of ELD1 in H is interesting since it is known to function related to defects in cell elongation and cellulose deficiency [69]. Together with the higher expressions of DEGs reported in cell size and stem elongation, VRN1, CNR6, mTOR, AP2-like, CCOAR, and BB in J compared to H clearly indicate their potential role in plant height in J [14,15,16,17,18]. Such changes in cell size and stem elongation are possible due to the presence of extensive cell wall biosynthesizing and modification-related genes [70]. Differential regulation of a large number of cell-wall-related genes in J and H is an indication of variations in cell-wall biosynthesis and modification at different levels. Therefore, these two plants may be useful for studying differential cell-wall biosynthesis in stems. Among the phytohormones, auxins stimulate cell elongation by increasing the extensibility of the cell wall [71], which is consistent with the differential expression of IAA biosynthesis and signal transduction genes and extensins (Figure 5). Studies have also reported that higher GA levels promote cell-wall thickness and change the deposition of cell-wall fibers in the xylem, thereby affecting organ growth [72]. GAs also enhance the anisotropy of cell expansion in maize leaves indicating their role in directional expansion of cells [73]. A recent study revealed that ABA and GA treatments can regulate phytohormonal signaling and cell wall fortification [74]. Our results that GA levels were higher in J and there were a large number of cell-wall-related (152) exclusively expressed genes in J suggest that there is a strong interaction between cell elongation by cell-wall modification under the influence of GA. Such an interaction was noticed by recent work in cotton where auxin promoted fiber elongation by enhancing GA biosynthesis [75]. Our data provides several candidate genes for examining their roles in the differential plant height in J and H under the influence of phytohormones. Specifically, we provide different candidate genes that can be characterized by using H and J to deeply investigate the cell size changes, cell number differences, and cell proliferation.

Transcription factors are known to play important roles in plant growth and development. Recent reports have highlighted that several transcription factors play important roles in controlling immunity, development, and plant architecture, in addition to growth [76]. This is obvious from the results that 734 TFs were differentially expressed between J and H (Table S6). The highest number of NAC TFs, among all, is indicative of their significant role in the differential growth of the two plants studied. They have been characterized in Arabidopsis to regulate secondary wall biosynthesis and hypocotyl elongation [77,78]. Moreover, differential expression of AP2/ERF-ERF, MYB-related, bHLH, and NAC have been reported in *Picea crassifolia* Kom during hypocotyl elongation inhibition in response to N6-benzyladenine (a cytokinin) [79]. This indicates that these TFs are associated with tissue elongation in other plants too. Therefore, our results provide a large number of candidate TFs that can be investigated for their role in plant tissue elongation under the action of phytohormones. Taken together, our data indicate that IAA-amino acid conjugates, GA, cytokinin, and JA are linked to the differential plant height in J and H. The differences in their levels are due to the differential expression of phytohormone biosynthesis and signal-transduction-related genes. The expression changes in these genes possibly affect the cell-wall biosynthesis/remodeling, cell size, number, and proliferation genes. Our laboratory has now successfully established a tissue culture system of *Z. schneideriana* and we plan to functionally characterize several genes highlighted in this study.

## 4. Material and Methods

### 4.1. Plant Material and Growth Conditions

The experiment was set up in a greenhouse at the College of Landscape Architecture, ChangSha, China. The temperature, humidity, and light/dark cycles were 25, 80%, and 16/8 h, respectively. Non-contaminated soil (natural soil used in this experiment) was collected from the top layer (0–20 cm) in a field station of the CSUFT campus, then dried and passed through a 5-mm sieve. The physicochemical properties of the soil included 12.13 g (total C) kg^−1^, 0.24 g (total N) kg^−1^, 0.16 g (total P) kg^−1^, pH 6.40, and Pb and Zn concentrations of 0.02 and 0.03 g kg^−1^, respectively.

The annual grafted branches of *ZS* and *ZS HenTianGao* during July 2021 were taken from Zhongnanlin Nursery, China (Figure 1a–c). The rootstocks for the experimental plants are the *ZS* (the common ZS that has large stems (J as shown in Figure 1). The scions for J and H were obtained from new buds that were grown in the same year of grafting—2021. The plant material was identified by Prof Xiaoling Jin. The voucher specimen is available at the Central South University of Forestry and Technology under the number: CSUFT-23-1X4D. One seedling was transplanted to a pot containing ~9 kg of air-dried and noncontaminated soil. After transplantation, the pot was kept in the greenhouse for 30 days. A total of 100 pots were used to grow the two types of grafted branches. Ten plants (five each for J and H) were randomly selected, and triplicate branch samples were taken from each of the four directions from these plants. While sampling, the branches were collected from different pots but from the same direction (southwest and northwest) of the trees. The triplicate branch samples were then subjected to the following analyses according to the described methodology.

### 4.2. Histological Analysis

The one-year-old branches of both H and J varieties were removed from the experimental plants, sliced with a surgical blade, stained with 0.05% toluidine blue, and observed under a Zeiss standard microscope (Carl Zeiss, Oberkochen, Germany) using CaseViewer 2.4 RTM v2.4.0.119028 x64 with CNV, resourced from the Wuhan Punais Testing Technology Co., Ltd., Wuhan National Biological Industry Based, Wuhan City, China.

### 4.3. Quantification of Endogenous Phytohormones

High-performance liquid chromatography (HPLC) grade reagents, acetonitrile, methanol Merck (Darmstadt, Germany), acetic acid, formic acid (Sigma-Aldrich, St. Louis, MO, USA), and MilliQ (Merck Millipore, Darmstadt, Germany) were used. The standards were purchased from Olchemim Ltd. (Olomouc, Czech Republic) and isoReag (Shanghai, China). Acetic acid and formic acid were bought from Sigma-Aldrich (St. Louis, MO, USA). The stock solutions of standards were prepared at the concentration of 1 mg/mL in MeOH. All stock solutions were stored at −20 °C. The stock solutions were diluted with MeOH to working solutions before analysis.

The freshly harvested triplicate branches of both varieties, J (*Zelkova schneideriana*) and H (HenTianGao), were frozen in liquid nitrogen and stored at −80 °C until further processed. For endogenous hormone level determination, the stored samples were ground into powder, weighed 50 mg in a 2 mL microtube, and dissolved in 1 mL methanol/water/formic acid (15:4:1, *v*/*v*/*v*) for extraction. For quantification, 10 μL internal standard mixed solution (100 ng/mL) was added into the extract as internal standards, followed by vortexing for 10 min and centrifugation for 5 min at 12,000 r/min and 4 °C. The supernatant was collected in new microtubes, dried by evaporation, and dissolved in 100 μL 80% methanol (*v*/*v*). The contents of microtubes were then filtered through a 0.22 μm membrane filter and used for LC-MS/MS analysis as described earlier [80].

The extracts were analyzed on a UPLC-ESI-MS/MS system (UPLC, ExionLC™ AD, https://sciex.com.cn/ (accessed on 2 December 2021); MS, Applied Biosystems 6500 Triple Quadrupole, https://sciex.com.cn/). The analytical conditions were set as reported earlier by Q Niu, Y Zong, M Qian, F Yang, and Y Teng [81]. These included LC: column, Waters ACQUITY UPLC HSS T3 C18 (100 mm × 2.1 mm i.d., 1.8 μm); solvent system, water with 0.04% acetic acid (A), acetonitrile with 0.04% acetic acid (B); gradient program, started at 5% B (0–1 min), increased to 95% B (1–8 min), 95% B (8–9 min), finally ramped back to 5% B (9.1–12 min); flow rate, 0.35 mL/min; temperature, 40 °C; injection volume: 2 μL. The linear ion trap and triple quadrupole (QQQ) scans were attained on a QQQ-linear ion trap mass spectrometer (QTRAP), QTRAP^®^ 6500+ LC-MS/MS System (AB Sciex LLC, Framingham, MA, USA), equipped with an ESI Turbo Ion-Spray interface, operating in both positive and negative ion mode and controlled by Analyst 1.6.3 software (AB Sciex LLC, Framingham, MA, USA) as reported earlier [82,83]. The ESI source operation parameters were as follows: ion source, ESI+/−; source temperature 550 °C; ion spray voltage (IS) 5500 V (+), −4500 V (−); curtain gas was set at 35 psi, respectively. Phytohormones were analyzed using scheduled multiple reaction monitoring (MRM). Data acquisitions were performed using Analyst 1.6.3 software (AB Sciex LLC, Framingham, MA, USA). Multiquant 3.0.3 software (AB Sciex LLC, Framingham, MA, USA) was used to quantify all metabolites. Mass spectrometer parameters including the de-clustering potentials (DP) and collision energies (CE) for individual MRM transitions were conducted with further DP and CE optimization. A specific set of MRM transitions were monitored for each period according to the metabolites eluted within this period. The identified compounds were annotated using KEGG compound database (http://www.kegg.jp/kegg/compound/ (accessed on 23 February 2022)) [84].

### 4.4. Transcriptome Analysis

High-quality total RNA was extracted from the freshly harvested triplicate samples (branches) of J and H by using the NEBNext^®^ UltraTM RNA Library Prep Kit Plant (New England, Biolabs Inc., Ipswich, MA, USA) following the manufacturer’s protocol [85]. The quality of the RNA was checked by NanoPhotometer spectrophotometer (IMPLEN, Westlake Village, CA, USA), while the integrity was checked on an Agilent 5400 Bioanalyzer (Agilent Technologies, Santa Clara, CA, USA). The mRNA was obtained as described in earlier studies [86] and first-strand cDNAs were synthesized by using random hexamers. Buffer and dNTPs were added to cDNAs, and double-stranded cDNA was obtained using DNA polymerase I, followed by purification by using AMPureXP beads. The purified ds cDNAs were end repaired, A-tailed, and added sequencing adapters, followed by fragment size selection. Finally, the PCR enrichment was carried out to obtain final cDNA libraries. The quality of the libraries was tested using Qubit2.0 for preliminary quantification and Agilent 2100 bioanalyzer for insert size detection of the libraries. The libraries were then sequenced on Illumina platform.

The raw reads were processed using fastp [87] to ensure the high accuracy of the subsequent analyses. For this, the reads with adapter were removed, the paired end reads with >10% (of the number of basis) N content were removed, and the paired end reads if the number of low-quality bases (Q ≤ 20) contained in the sequencing exceeded 50% of the number of bases in the read were removed. The distribution of the sequencing error and GC content was determined, and clean reads were obtained and used for further analyses. Clean reads were assembled using Trinity [88]. Hierarchical clustering of transcripts was conducted in Corset [89]. The unigenes generated by Corset were used as a reference for DEG analyses. For functional annotation, DIAMOND [90] BLASTX software (https://blast.ncbi.nlm.nih.gov/; accessed on 21 April 2022) was used to compare the unigene sequences with the KEGG [91], NR [92], Swiss-Prot [93], GO [94], COG/KOG [95], and Trembl databases, and after predicting the amino acid sequences, HMMER web server [96] was used to compare them with the Pfam database to obtain annotation information. Bowtie2 [97] in RSEM [98] was also used for the gene expression quantification. Fragments Per Kilobase of transcripts per Million fragments mapped (FPKM) were used as an indicator of transcript/gene expression. The FPKM values were used to determine the overall distribution of gene expression, Pearson’s Correlation Coefficient (PCC), and Principal Component Analysis (PCA). DESeq2 [99] was used for determining differential gene expression between the two sample types, and differentially expressed genes (DEGs) were screened based on false discovery rate (FDR) < 0.05 and |log2 Fold Change| ≥ 1. The KEGG and GO pathway enrichment analyses of the DEGs were completed using KOBAS-2.0 [100]. The transcription factors (TFs) prediction was completed using iTAK [101] against PlnTFDB [102] and PlantTFDB [103] databases. 

### 4.5. qRT-PCR Analyses

Nineteen *Z. schneideriana* genes showing interesting expression profiles between J and H were selected for qRT-PCR analysis. First-strand cDNA was synthesized from 100 ng total RNA by using a high-capacity cDNA reverse transcription kit (Applied Biosystem, Carlsbad, CA, USA). This will be updated based on the provided PCR profiles and primer sequences. Primers for the selected genes were designed using Primer3 (http://frodo.wi.mit.edu/primer3/ (accessed on 21 April 2022)); (Table S7). The *Actin2* gene was used as an internal control. The reactions were carried out with a final volume of 20 μL as reported earlier [6]. The reactions were carried out on a Rotor-Gene 6000 machine (Qiagen, Shanghai, China). The reactions were carried out in triplicate. Relative gene expression was computed according to the method described by [104]. Correlation between the FPKM values and relative gene expression data was computed in R using the cor function.

## 5. Conclusions

We report comparative phytohormone profiles and transcriptome sequencing of *Z. schneideriana* and its dwarf variety HenTianGao. Based on the combined results of endogenous phytohormone levels and the transcriptome changes, we suggest several potential pathways that could be associated with the differential height in the two varieties. Noticeably, we conclude that the conversion of auxin to its conjugates, OxIAA and IAA-Glc, is higher in HanTianGao compared to *Z. schneideriana*, leading to reduced detection of IAA to the IAA-signaling genes. Similarly, the comparable levels of GA19, trans Zeatin, jasmonic acid, and jasmonic acid isoleucine in *Z. schneideriana* and HanTianGao are a potential reason for the differential height of the two varieties. We also conclude that, other than phytohormones mentioned above, cell size, cell number, cell proliferation, and stem-elongation-related genes (and pathways) play significant roles in the observed phenotypes of HanTianGao and *Z. schneideriana*. Our results provide important data for understanding the potential molecular regulatory mechanisms for the differential plant height in *Z. schneideriana.*

## Data Availability

The raw RNA-seq datasets generated in this study can be found at NCBI SRA under the project number: PRJNA974708 [https://www.ncbi.nlm.nih.gov/sra/?term=PRJNA974708; accessed on 16 November 2023].

## Supplementary Materials

The following supporting information can be downloaded at: https://www.mdpi.com/article/10.3390/ijms242316902/s1.
