# Supplementary figures and images for: Comparative Transcriptome Sequencing and Endogenous Phytohormone Content of Annual Grafted Branches of Zelkova schneideriana and Its Dwarf Variety HenTianGao
Source: Int J Mol Sci. 2023 Nov 29;24(23):16902. doi: 10.3390/ijms242316902 (PMC10706849; doi:10.3390/ijms242316902)

## Slide 1
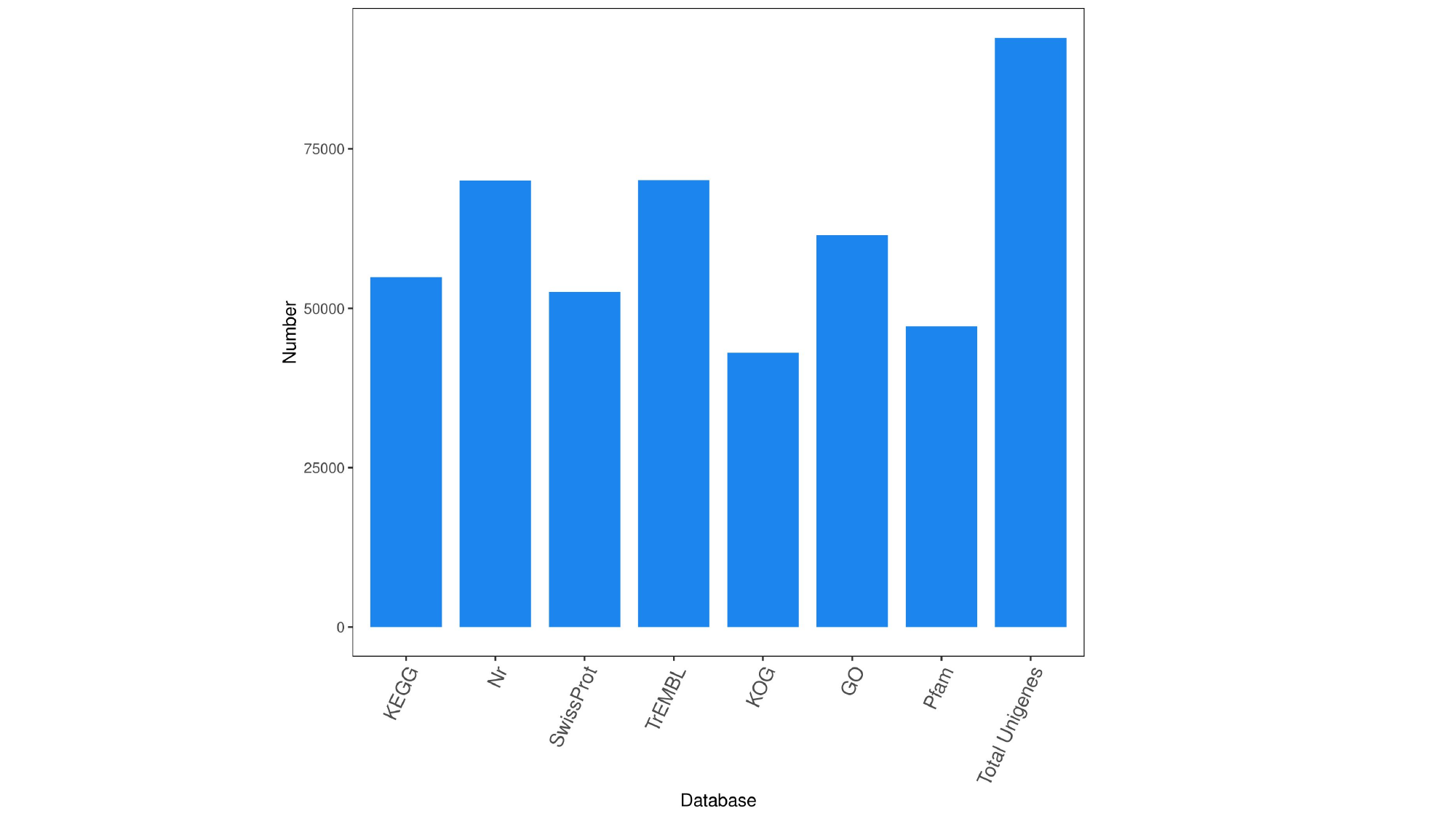

Supplement: Supplementary file 1 [file ijms-24-16902-s001.zip › Figure S1V1.pptx]

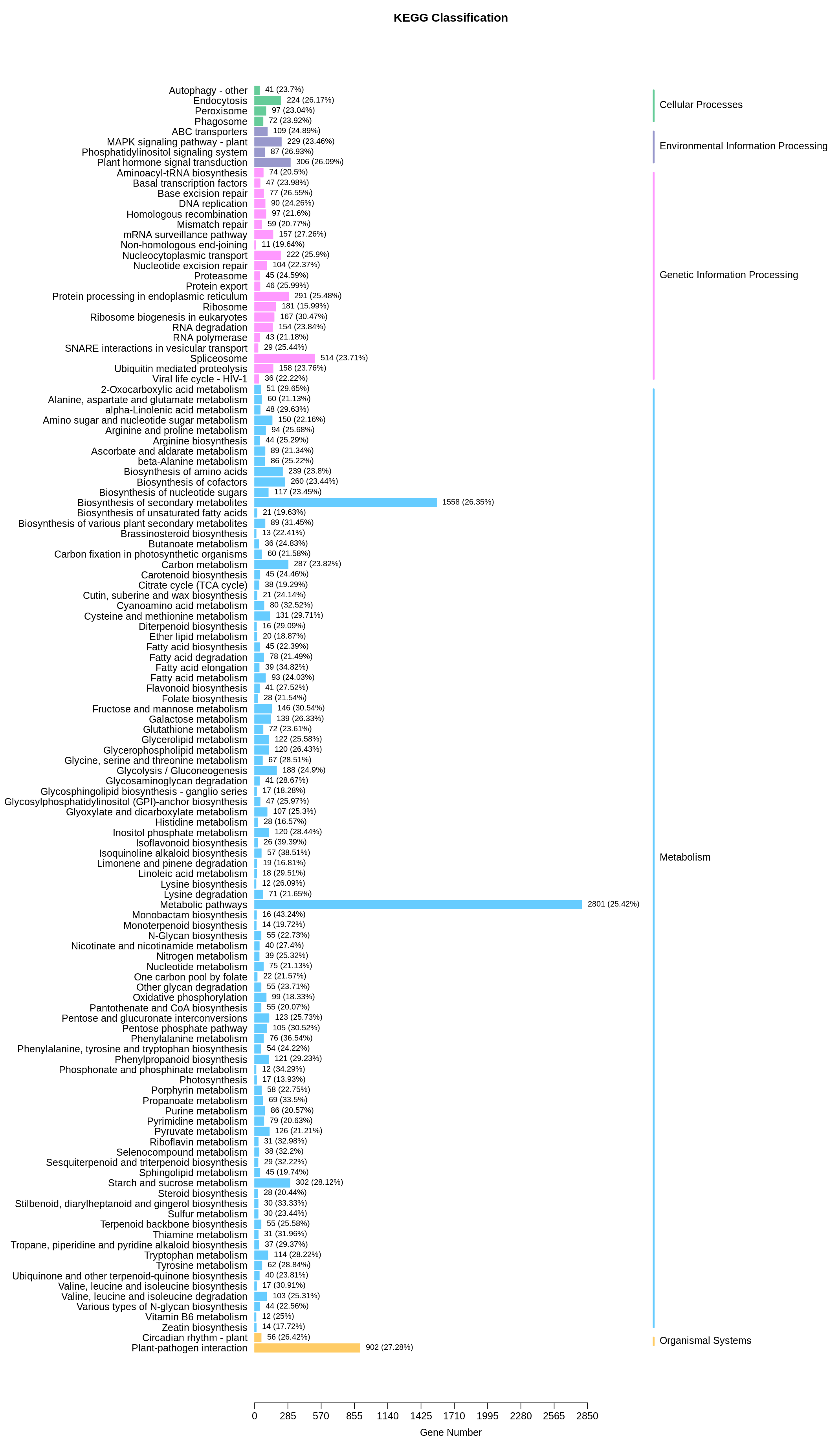

Supplement: Supplementary file 1 [file ijms-24-16902-s001.zip › Figure S2aV1.png]

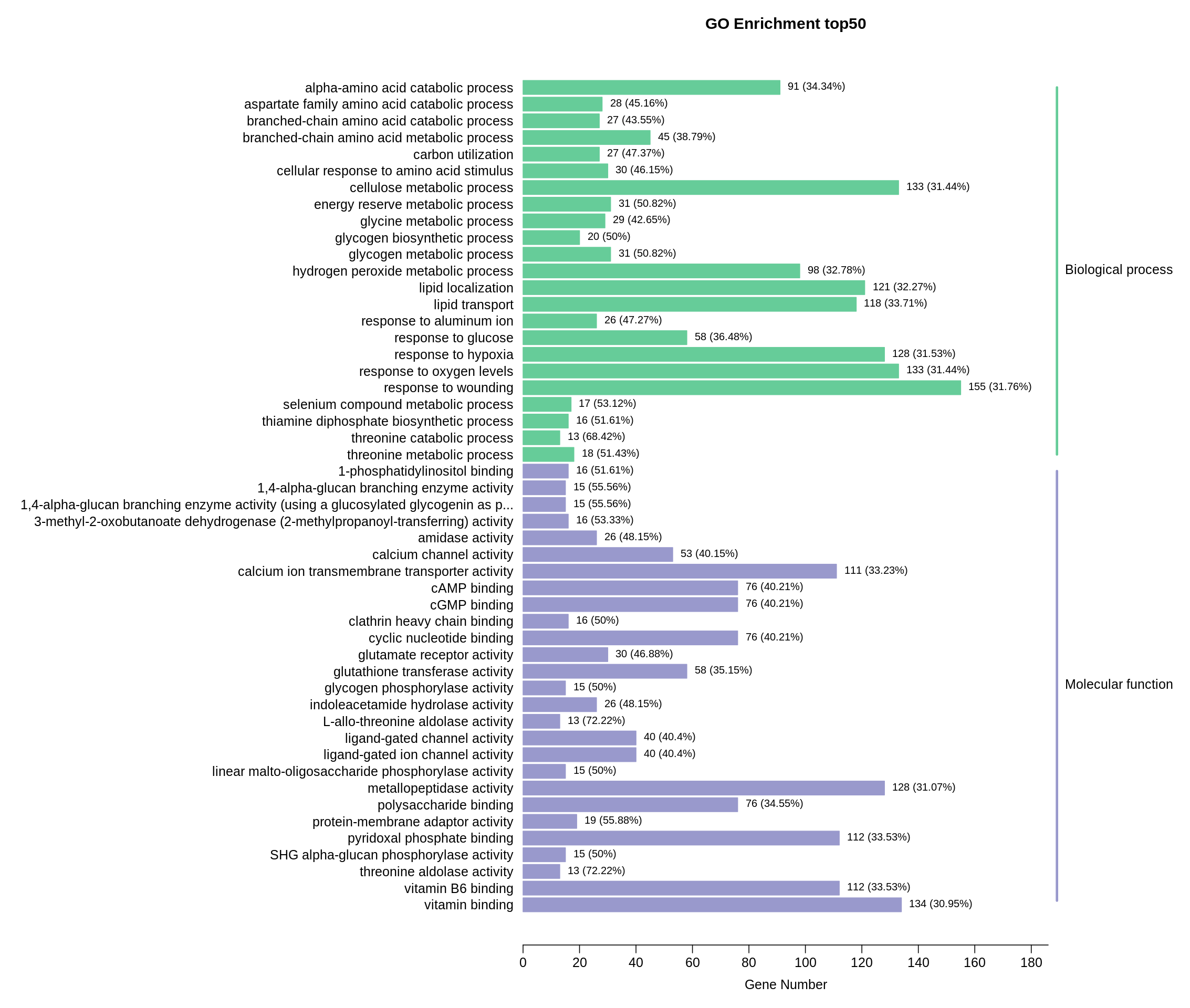

Supplement: Supplementary file 1 [file ijms-24-16902-s001.zip › Figure S2bV1.png]
